# Dietary management improves sleep quality in patients with metabolic syndrome: the mediating roles of metabolic, inflammatory, and oxidative stress changes

**DOI:** 10.3389/fnut.2025.1712215

**Published:** 2025-12-12

**Authors:** Junxiang Cheng, Qia Liu, Zhouli Qiu, Mengyi Pan, Jie Song, Yu Zhao, Niuniu Dong, Suping Li, Shifan Han

**Affiliations:** 1 Department of Psychiatry, The First Hospital of Shanxi Medical University, Taiyuan, China; 2 Shanxi Provincial Clinical Medical Research Center for Mental and Psychological Disorders (Depression), Taiyuan, China; 3 Shanxi Key Laboratory of Artificial Intelligence Assisted Diagnosis and Treatment for Mental Disorder, Taiyuan, China; 4 School of Nursing, Shanxi Medical University, Taiyuan, China; 5 The First Hospital of Shanxi Medical University, Taiyuan, China

**Keywords:** metabolic syndrome, sleep disorders, dietary intervention, inflammation, oxidative stress, Family Nurse Dietary Therapy

## Abstract

**Background:**

Sleep disorders frequently co-occur with metabolic syndrome (MetS), yet effective strategies targeting both conditions remain limited. Inflammation and oxidative stress represent shared mechanisms, suggesting dietary management as a promising dual-target intervention. This study aimed to evaluate whether structured dietary management could improve sleep quality and metabolic, inflammatory, and oxidative stress parameters in patients with MetS.

**Methods:**

We conducted a single-arm prospective interventional study including 158 patients with MetS and sleep disorders [Pittsburgh Sleep Quality Index (PSQI) > 7] between August and October 2024. Participants received a structured dietary management program. Clinical characteristics, metabolic parameters, and inflammatory and oxidative stress biomarkers were assessed before and after intervention. Paired tests evaluated pre–post changes, and stepwise multivariate linear regression was performed to identify independent predictors of sleep quality.

**Results:**

Dietary intervention significantly improved liver enzymes, lipid profile (triglycerides, LDL-C, HDL-C), glucose metabolism (fasting glucose, fasting insulin, HOMA-IR), and uric acid levels (all *P* < 0.05). TNF-α and hsCRP were markedly reduced (*P* < 0.001), while IL-6 showed a non-significant trend (*P* = 0.075). Oxidative stress improved, with lower MDA and higher SOD levels (*P* < 0.05). Regression analysis identified smoking status and insulin resistance as independent predictors of PSQI scores, underscoring the interplay between lifestyle factors and metabolic dysfunction in sleep health.

**Conclusion:**

Structured dietary management improves metabolic, inflammatory, and oxidative stress profiles while enhancing sleep quality in patients with MetS. The findings highlight dietary and lifestyle modifications as integral to comprehensive management strategies for MetS with sleep disturbances.

## 1 Introduction

Metabolic syndrome (MetS) is a cluster of metabolic abnormalities characterized by central obesity, insulin resistance, hypertension, hyperglycemia, and dyslipidemia, representing a major global public health challenge. A meta-analysis of 147 cross-sectional studies covering 120 million individuals reported a global prevalence of 25.4% in adults, with rates of 24.8% in men and 25.4% in women, and the gap between high- and low-income countries is narrowing (1). Sleep disorders are highly prevalent among individuals with MetS, and evidence suggests a bidirectional interaction between the two conditions (2). MetS arises from complex interactions between genetic predisposition and environmental factors, with heritability estimated at 10%–30% (3), indicating that genetic factors only partly explain its pathogenesis. Sedentary lifestyle and poor dietary habits, such as excessive intake of energy-dense, fatty, and sugary foods, contribute to obesity and insulin resistance, thereby exacerbating metabolic abnormalities. Conversely, sleep disturbances can elevate cortisol secretion from the adrenal cortex, leading to increased caloric intake and fat accumulation. Thus, metabolic dysregulation may impair sleep through inflammation and neuroendocrine mechanisms, while insufficient or disrupted sleep further aggravates insulin resistance and lipid abnormalities (4, 5).

Despite the established interplay between metabolism and sleep, comprehensive intervention strategies targeting both outcomes in patients with MetS remain limited. Existing dietary interventions primarily focus on metabolic improvements, and systematic evaluation of their impact on sleep quality is scarce. Our research team has previously demonstrated that dietary management guided by the Family Nurse Dietary Therapy, a structured nutrition care model integrating nursing and dietary strategies, can significantly reduce BMI, improve clinical outcomes, and enhance quality of life in chronic disease populations (6–8). The recent observational and interventional studies indicating a bidirectional relationship between MetS and poor sleep quality, where diets rich in anti-inflammatory and antioxidant compounds are biologically plausible for improving metabolic risk factors, reducing inflammation, and enhancing sleep phenotypes (9). Although outcomes from dietary interventions vary, the overall trend supports the superiority of whole-food, plant-based dietary patterns over single-nutrient supplementation (10).

Dietary interventions may offer dual benefits: obesity induces systemic oxidative stress, exacerbating metabolic abnormalities and sleep disturbances (11), while insufficient sleep can further impair insulin sensitivity, elevate cortisol levels, and promote weight gain (12). For instance, a study by Hung et al. in Taiwan revealed that patients with MetS had higher global PSQI scores and an increased risk of poor sleep, with hyperglycemia and low HDL-C independently associated with PSQI, and low HDL-C being a strong predictor of poor sleep quality (13).

Although metabolic dysfunction, inflammation, oxidative stress, and sleep disturbance are closely interrelated, few interventional studies have evaluated how dietary management affects these pathways in patients with MetS. This study examined whether a structured dietary program could improve sleep quality, metabolic profiles, and inflammatory and oxidative stress markers, aiming to clarify the potential of dietary management as a comprehensive, non-pharmacological approach for MetS-related sleep disorders.

## 2 Materials and methods

### 2.1 Study design and participants

This study adopted a single-arm, self-controlled design and recruited adult patients diagnosed with metabolic syndrome (MetS) from community settings in Taiyuan Shanxi between August and October 2024. The study was approved by the Ethics Committee of the First Hospital of Shanxi Medical University [(2022) (K044)] and registered at the Chinese Clinical Trial Registry (ChiCTR2200066645). All participants provided written informed consent prior to enrollment.

Inclusion criteria: Meeting the 2017 diagnostic criteria for MetS of the Chinese Society of Endocrinology:Central obesity: waist circumference ≥ 90 cm in men or ≥ 85 cm in women; Hypertension: blood pressure ≥ 130/85 mmHg or receiving anti-hypertensive treatment; Hypertriglyceridemia: fasting triglycerides ≥ 1.70 mmol/L; Low HDL-C: fasting high-density lipoprotein cholesterol < 1.04 mmol/L for men or < 1.29 mmol/L for women; Hyperglycemia: fasting plasma glucose ≥ 6.1 mmol/L and/or 2-h OGTT ≥ 7.8 mmol/L, or previously diagnosed diabetes under treatment. Diagnosis requires the presence of ≥3 criteria; Age ≥ 18 years; Pittsburgh Sleep Quality Index (PSQI) score ≥ 7; No participation in other clinical trials recently; Voluntary participation with signed informed consent.

Exclusion criteria: Diagnosed psychiatric disorders other than sleep disorders; Severe hepatic or renal dysfunction, malignancy, pregnancy, or lactation; Allergy to trial-related foods (e.g., buckwheat, legumes); Regular use of sedative-hypnotic drugs or other medications for sleep disorders within 2 weeks prior to the study that could not be discontinued during the trial; Recent or prior participation in structured non-pharmacological interventions targeting sleep or metabolic diseases (e.g., dietary therapy, cognitive behavioral therapy).

Sample size calculation: The primary endpoint was the change in Pittsburgh Sleep Quality Index (PSQI) total score. Using the paired *t*-test formula: $n={\left(\frac{Z_{\alpha /2}+Z_{\beta }}{\Delta /s_{d}}\right)}^{2}$ with α = 0.05 (two-sided) and 80% power, the minimally clinically important difference (MCID) was set at two points, and the standard deviation of paired differences (s<sub>d</sup>) was estimated at 1–4 points based on prior studies (14, 15). The calculated sample size was 32, increased to 40 to account for 20% attrition. Ultimately, 158 participants completed the study, providing >0.99 power to detect clinically meaningful changes.

### 2.2 Intervention materials and protocol

#### 2.2.1 Intervention materials

Two types of dietary supplements were used during the intervention. Plant-based protein compound powder: a specially formulated product primarily composed of soy protein isolate (protein content ≥ 90%). Herbal infusion beverage: a tea substitute processed with a proprietary method, containing tartary buckwheat, goji berries, and other plant-derived bioactive ingredients.

#### 2.2.2 Intervention protocol

The dietary intervention was designed using body composition analysis, dietary exchange units, and nutritional software to generate individualized meal plans. Adjustments were made weekly based on participants’ feedback and adaptability. The total intervention period lasted 6 weeks. The specific steps were as follows:

(1) Assessment phase: Baseline information was collected, and participants’ dietary habits and preferences were evaluated using a 3-day, 24-h dietary recall; (2) To ensure gradual and safe metabolic improvement, a moderate caloric restriction protocol was applied. Drawing on our previous research (6–8), a weight-based energy prescription was implemented (25 kcal/kg/day for BMI 24–27.9, 22 kcal/kg/day for BMI 28–34.9, and 20 kcal/kg/day for BMI ≥ 35), with minimal absolute intake thresholds of 1,200 kcal/day for women and 1,500 kcal/day for men. This individualized range was established to accommodate interindividual variability in baseline body weight, habitual physical activity, and occupational energy demands. (3) The dietary macronutrient distribution was standardized across individualized menus as follows: Carbohydrates: 45%–50% of total energy, primarily from whole grains, legumes, fruits, and vegetables; Protein: 20%–25% (to reduce the proportion of refined carbohydrates, a protein-based compound powder was provided as a partial replacement for staple foods at each meal); Fat: 25%–30%, primarily unsaturated fatty acids from nuts, seeds, and plant oils. At the same time, the intake of whole grains, vegetables, fruits, and dietary fiber was encouraged, whereas red meat and high-fat, high-sugar foods were limited; (4) The glycemic index (GI) of foods and their potential anti-inflammatory properties were considered in the design; (5) To ensure consistency in fluid intake and minimize potential confounding from sugar-sweetened or caffeinated beverages, patients consumed a plant-based infusion during the day instead of plain water or commercial beverages.

Cooking methods: Low-fat preparation methods such as steaming, boiling, blanching, and cold-mixing were recommended, while deep-frying was discouraged.

Follow-up and adjustment: Participants were monitored and supported through a mobile application, with timely adjustments made to the dietary plan as needed.

During the intervention, participants were instructed to maintain their usual physical activity levels and medication regimens. Any necessary medication adjustments were promptly communicated with the study team.

### 2.3 Outcome measures

Baseline demographic and clinical characteristics were collected, including sex, age, height, weight, BMI, blood pressure, and medical history. Laboratory tests were performed before and after the intervention, covering blood glucose, blood lipids, liver and kidney function, insulin, and the homeostasis model assessment of insulin resistance (HOMA-IR). Inflammatory markers (IL-6, TNF-α, CRP) and oxidative stress parameters [malondialdehyde (MDA) and superoxide dismutase (SOD)] were also measured.

Sleep quality was assessed using the Pittsburgh Sleep Quality Index (PSQI), developed by Buysse et al. in 1989. The PSQI has been widely validated and shows significant correlation with polysomnography-based sleep assessments. It is one of the most commonly used clinical tools for evaluating sleep quality in psychiatric and neurological settings, both internationally and in China. In Chinese populations, the PSQI has demonstrated strong test–retest reliability and validity in adults with sleep disorders. The scale consists of 19 self-rated items and five clinician-rated items, though only 18 self-rated items are included in the scoring. These items cover seven components, each scored on a 0–3 scale, with a total score ranging from 0 to 21. Higher scores indicate poorer sleep quality. In this study, a PSQI score > 7 was used to define the presence of sleep disturbance. Reported psychometric properties of the Chinese version include a test–retest reliability of 0.9994, split-half reliability coefficient of 0.842, and a Cronbach’s α of 0.845 (16).

### 2.4 Statistical analysis

All statistical analyses were performed using SPSS version 26.0 (IBM Corp., Armonk, NY, United States). Continuous variables were tested for normality. Normally distributed data are expressed as mean ± standard deviation, and non-normally distributed data are expressed as median (interquartile range). Paired-sample *t*-tests or Wilcoxon signed-rank tests were used to compare pre- and post-intervention data.

In addition, to further explore the factors associated with improvements in sleep quality, we calculated the difference values (post-intervention minus pre-intervention) for relevant variables that showed statistically significant changes after dietary intervention. Linear regression analyses were then conducted using the change in sleep quality score (ΔPSQI) as the dependent variable. Multicollinearity was assessed using tolerance and variance inflation factor (VIF). A *p*-value < 0.05 was considered statistically significant. Considering potential multicollinearity among variables in the multivariate linear regression, we further employed a stepwise linear regression approach to identify the most parsimonious model.

### 2.5 Quality control

To ensure the accuracy and reliability of the study, several quality control measures were implemented. First, all research staff underwent systematic training to standardize their understanding of the study protocol, assessment tools, and data collection procedures. Second, collected data were processed following standardized protocols, including unit harmonization, data cleaning, and handling of missing values, to guarantee precision and consistency. Third, adherence to the dietary plan was monitored and supported through a WeChat-based mobile mini-program specifically developed by our research team in collaboration with software engineers. Participants were trained to use the program during enrollment, which enabled them to record daily meals and check in at least 5 days per week. The application provided real-time feedback to both participants and researchers, allowing timely dietary adjustments. In addition to app-based monitoring, research staff maintained regular communication with participants via WeChat messages to address difficulties, encourage compliance, and minimize dropout rates. Compliance was assessed as the proportion of weekly check-ins completed; participants with < 80% adherence were flagged for additional follow-up. No participant withdrew due to dietary intolerance, and overall adherence exceeded 85%.

## 3 Results

### 3.1 Baseline characteristics

A total of 158 patients with metabolic syndrome (MetS) and comorbid sleep disturbances were enrolled in this study, including 63 males and 95 females. The mean age of participants was 48.51 ± 14.86 years. All patients met the diagnostic criteria for MetS, and their PSQI scores were greater than seven. Baseline demographic and lifestyle characteristics, including height, weight, educational level, smoking and alcohol consumption, physical activity intensity, and dietary preferences, are summarized in Table 1.

**TABLE 1:** Baseline characteristics of patients with metabolic syndrome and sleep disturbances (*n* = 158).

Variable	Category/value	*N* = 158	%
➀➁➂	/	19	**12.0**
➀➁➃	/	29	**18.35**
➀➁➄	/	19	**12.0**
➀➁➂➃	/	58	**36.71**
➀➁➃➄	/	12	**7.60**
➀➁➂➃➄	/	21	**13.30**
Sex	Male	63	39.9
	Female	95	60.1
Age (years)	48.51 ± 14.86	158	100
Height (cm)	164.21 ± 8.43	158	100
Weight (kg)	73.61 ± 14.07	158	100
Education level	Primary school	1	0.63
	Junior high school	21	13.29
	Secondary/High school	12	7.59
	Junior college	56	35.44
	Bachelor’s degree	54	34.18
	Postgraduate	14	8.86
Smoking status	Current smoker	26	16.5
	Non-smoker	132	83.5
Alcohol intake	Yes	40	25.3
	No	118	74.7
Physical activity	Light work	124	78.5
	Moderate work	34	21.5
Dietary preference	Meat-based	62	39.2
	Vegetarian	65	41.1
	Mixed	31	19.6

➀ Central obesity: waist circumference ≥ 90 cm in men or ≥ 85 cm in women; ➁ Hypertension: blood pressure ≥ 130/85 mmHg or receiving anti-hypertensive treatment; ➂ Hypertriglyceridemia: fasting triglycerides ≥ 1.70 mmol/L; Low HDL-C: ➃ Fasting high-density lipoprotein cholesterol < 1.04 mmol/L for men or < 1.29 mmol/L for women; ➄ Hyperglycemia: fasting plasma glucose ≥ 6.1 mmol/L and/or 2-h OGTT ≥ 7.8 mmol/L, or previously diagnosed diabetes under treatment. Bold values indicate that difference is statistically significant.

### 3.2 Comparison of sleep quality, body weight, and BMI before and after intervention

After 6 weeks of dietary management, the PSQI score of patients was significantly reduced compared with baseline (14.09 ± 5.75 vs. 10.15 ± 6.40, *t* = 9.968, *P* < 0.001), indicating a marked improvement in sleep quality. In addition, body weight showed a significant decrease from 73.62 ± 14.07 to 65.25 kg (59.00, 76.07) (*Z* = −10.90, *P* < 0.001), and BMI was significantly reduced from 27.16 ± 3.52 to 25.21 ± 3.18 (*t* = 26.11, *P* < 0.001). These findings suggest that the structured dietary management program not only improved sleep quality but also exerted beneficial effects on weight control and BMI reduction in patients with metabolic syndrome and comorbid sleep disorders (Table 2).

**TABLE 2:** Comparison of sleep quality, body weight, and body mass index (BMI) before and after dietary management (*n* = 158).

Variable	*n*	Pre-intervention (Mean ± SD/M[P25, P75])	Post-intervention (Mean ± SD/M[P25, P75])	Test statistic	*P*-value
PSQI score	158	14.09 ± 5.75	10.15 ± 6.40	9.968	**<0.001**
Body weight (kg)	158	73.62 ± 14.07	65.25 (59.00, 76.07)	−10.90	**<0.001**
BMI (kg/m^2^)	158	27.16 ± 3.52	25.21 ± 3.18	26.11	**<0.001**

Paired-sample *t*-tests and Wilcoxon signed-rank tests were used to compare pre- and post-intervention data. Bold values indicate that difference is statistically significant.

### 3.3 Comparison of biochemical parameters before and after intervention

Biochemical assessments revealed significant improvements following the dietary intervention. Fasting plasma glucose (FPG), triglycerides (TG), total cholesterol (TC), low-density lipoprotein cholesterol (LDL-C), and the homeostasis model assessment of insulin resistance (HOMA-IR) all showed significant reductions compared with baseline (*P* < 0.05 or *P* < 0.001). In contrast, high-density lipoprotein cholesterol (HDL-C) levels increased significantly (*P* < 0.05). These findings indicate that the dietary management program contributed to favorable regulation of glucose and lipid metabolism and alleviation of insulin resistance in patients with metabolic syndrome and comorbid sleep disorders (Table 3).

**TABLE 3:** Comparison of biochemical parameters before and after intervention.

Parameter	*n*	Pre- intervention	Post- intervention	Statistic	*P*-value
ALT (U/L)	158	23.00 (14.00, 38.00)	20.00 (14.00, 26.00)	−4.09	**<0.001**
AST (U/L)	158	21.00 (17.00, 26.00)	20.00 (17.00, 23.00)	−2.33	**0.020**
Urea (mmol/L)	158	4.50 (3.88, 5.60)	4.89 ± 1.58	−1.21	0.223
Serum creatinine (μmol/L)	158	63.37 ± 14.63	62.83 ± 13.74	0.55	0.583
Uric acid (μmol/L)	158	362.45 ± 102.63	300.00 (246.75, 367.25)	−7.37	**<0.001**
Triglycerides (TG, mmol/L)	158	1.95 (1.31, 2.67)	1.35 (0.92, 1.95)	−7.79	**<0.001**
Total cholesterol (TC, mmol/L)	158	4.75 ± 1.10	4.53 ± 0.88	2.887	**0.004**
HDL-C (mmol/L)	158	1.17 ± 0.37	1.27 ± 0.40	−3.093	**0.002**
LDL-C (mmol/L)	158	2.73 ± 0.86	2.56 ± 0.73	1.57	**0.005**
Fasting plasma glucose (FPG, mmol/L)	158	5.08 (4.54, 6.19)	4.80 (4.27, 5.50)	−6.508	**<0.001**
Fasting insulin (μIU/mL)	158	11.99 (7.79, 18.48)	9.04 (6.09, 15.03)	−6.973	**<0.001**
HOMA-IR	158	2.18 (1.43, 4.03)	1.43 (0.76, 2.75)	−7.904	**<0.001**

Paired-sample *t*-tests or Wilcoxon signed-rank tests were used to compare pre- and post-intervention data. Bold values indicate that difference is statistically significant.

### 3.4 Comparison of inflammatory and oxidative stress markers before and after intervention

The assessment of inflammatory and oxidative stress markers before and after the intervention indicated that IL-6 levels did not change significantly (*P* = 0.075). However, pro-inflammatory markers including tumor necrosis factor-alpha (TNF-α) and high-sensitivity C-reactive protein (hsCRP) showed significant reductions after the intervention (*P* < 0.05 or *P* < 0.001), suggesting that the dietary management program effectively mitigated systemic inflammation. Meanwhile, malondialdehyde (MDA), an indicator of oxidative stress, decreased significantly (*P* < 0.001), whereas superoxide dismutase (SOD) levels increased (*P* < 0.05), further confirming the beneficial effect of dietary intervention on oxidative stress. The dual improvement in inflammation and oxidative stress may provide an important physiological basis for alleviating metabolic syndrome and sleep disturbances in these patients (see Table 4).

**TABLE 4:** Comparison of inflammatory and oxidative stress markers before and after intervention.

Parameter	*n*	Pre- intervention	Post-intervention	Statistic	*P*-value
hsCRP (mg/L)	158	1.50 (0.60, 3.08)	0.70 (0.20, 1.73)	−5.533	**<0.001**
IL-6 (pg/mL)	158	4.20 (2.83, 9.75)	4.19 (2.30, 9.44)	−1.782	0.075
TNF-α (pg/mL)	158	4.29 (3.58, 5.33)	3.93 (3.16, 5.18)	−3.731	**<0.001**
MDA (μmol/L)	158	1.87 (1.69, 2.07)	1.54 ± 0.26	−9.521	**<0.001**
SOD (U/mL)	158	173.15 ± 21.32	181.93 ± 18.93	−5.56	**<0.001**

Paired-sample *t*-tests or Wilcoxon signed-rank tests were used to compare pre- and post-intervention data. Bold values indicate that difference is statistically significant.

### 3.5 Linear regression analysis of factors influencing changes in sleep quality

As shown in Table 5, higher BMI reduction was significantly associated with greater improvements in sleep quality (*B* = 4.249, *p* = 0.001). In the multivariate model, smoking status (*B* = 4.360, *p* < 0.001) and HOMA-IR reduction (*B* = 2.216, *p* = 0.024) remained significant positive predictors of improved sleep quality, while LDL-C reduction was inversely associated (*B* = −1.226, *p* = 0.010). Variance inflation factors (VIF < 1.01) indicated no collinearity concerns. Neither age nor gender was significantly associated with changes in PSQI scores after the dietary intervention (both *p* > 0.05).

**TABLE 5:** Linear regression analysis of factors influencing changes in sleep quality.

Variable	*B*	SE	Beta	*t*	*P*-value	Tolerance	VIF
Constant	1.351	0.412	–	3.277	0.002	–	–
BMI	4.249	1.195	0.490	3.555	**0.001**	1.000	1.000
Smoking	4.360	1.112	0.503	3.922	**0.000**	0.999	1.001
LDL-C	−1.226	0.454	−0.346	−2.700	**0.010**	0.999	1.001
HOMA-IR	2.216	0.834	1.373	2.657	**0.024**	0.063	1.001

Linear regression analyses were conducted using the change in sleep quality score (ΔPSQI) as the dependent variable. Bold values indicate that difference is statistically significant.

These results suggest that improvements in sleep quality after dietary management were partly mediated by changes in metabolic parameters and lifestyle-related factors, particularly weight status, smoking, lipid metabolism, and insulin resistance.

## 4 Discussion

This study revealed a high prevalence of sleep disturbances among patients with metabolic syndrome (MetS), closely associated with metabolic dysregulation, systemic inflammation, and oxidative stress. We implemented a structured dietary management program emphasizing energy balance, macronutrient optimization, and plant-based food inclusion in patients diagnosed with MetS and concurrent sleep disturbances. After 6 weeks of intervention, sleep quality, as measured by PSQI scores, improved significantly, BMI decreased, and biochemical markers including blood lipids, glucose, liver and kidney function, HOMA-IR, hsCRP, MDA, TNF-α, and SOD showed notable improvements, whereas IL-6 did not change significantly. These findings suggest that dietary intervention not only optimizes energy metabolism but may also confer synergistic benefits through the “inflammation–oxidative stress–sleep” axis.

Consistent with our findings, Toğuç et al. demonstrated that dietary intervention improved sleep quality and reduced inflammatory markers (17). Moreover, recent evidence indicates that plant-based dietary patterns may improve sleep and metabolic health, reinforcing the potential of dietary strategies in managing sleep disturbances among individuals with metabolic dysfunction (18). Structured dietary management can effectively improve both sleep quality and metabolic parameters. Previous research has shown that Mediterranean and DASH diets—rich in unsaturated fats and fiber—reduce inflammation (e.g., CRP), support weight management, enhance antioxidant capacity, and improve insulin resistance (19). Ketogenic diets (high-fat, low-carbohydrate) also show benefits in inducing ketosis, improving insulin sensitivity, lowering triglycerides, and enhancing fat oxidation, although their long-term adherence and potential nutrient deficiencies remain limitations (20). The dietary approach used in this study focused not only on total energy and macronutrient balance but also on incorporating plant-based proteins and bioactive plant components with antioxidant and anti-inflammatory properties. This comprehensive approach may explain the significant effects observed within 6 weeks.

The regression analysis further highlighted that reductions in BMI and improvements in insulin resistance were significant predictors of enhanced sleep quality, suggesting that weight loss and improved glucose–insulin homeostasis may play central roles in the bidirectional relationship between metabolic syndrome and sleep. Optimizing protein intake, particularly from plant-based sources, can improve nutritional quality while reducing intake of saturated fats, supporting weight loss and BMI reduction (21). The intervention also included bioactive plant components derived from whole-food sources, which may contribute to improvements in metabolic and oxidative stress markers. Similar research has shown that increasing protein intake in MetS patients can lead to significant weight loss and improved sleep efficiency. BMI reduction may improve insulin resistance and inflammation, potentially creating a positive feedback loop with enhanced sleep quality. Weight control has been repeatedly shown to improve both objective and subjective sleep outcomes (22). In this study, BMI reduction may act as a mediator through multiple pathways: lowering systemic low-grade inflammation and oxidative stress, improving insulin sensitivity and lipid profiles, and mitigating risks of airway collapse and sleep fragmentation.

Although IL-6 levels did not significantly decrease in this study, hsCRP and TNF-α were markedly reduced, SOD activity increased, and MDA levels declined. These effects may be related to the dietary plan’s inclusion of polyphenol-rich plant foods, which can enhance endogenous antioxidant enzyme activity (e.g., SOD) and reduce lipid peroxidation (e.g., MDA). Systematic reviews and meta-analyses indicate that polyphenols consistently increase SOD activity and reduce MDA levels, though their effects on IL-6, TNF-α, and CRP show heterogeneity (23). This aligns with our findings of significant hsCRP and TNF-α reductions, but minimal IL-6 change. Furthermore, evidence suggests that polyphenol-rich plant foods improve body composition and metabolic profiles by modulating gut microbiota, reducing oxidative stress, and suppressing inflammation (24). Adjusting dietary structure to include these components may therefore contribute to improvements in sleep quality (25).

The improvement in sleep quality in this study may be attributed to multiple factors. Weight and metabolic improvements, including reduced BMI, improved insulin resistance (HOMA-IR), and optimized blood glucose and lipid levels, may alleviate sleep disturbances such as sleep apnea and enhance overall sleep quality. Dietary optimization, including increased intake of high-fiber, low-glycemic, and polyphenol-rich foods, may stabilize blood glucose, reduce fluctuations, and enhance satiety, facilitating sleep initiation and maintenance. Reduced inflammation likely contributes directly to improved sleep, as elevated hsCRP and TNF-α are commonly associated with sleep disturbances (26). Likewise, decreased oxidative stress may promote neuroendocrine stability and support sleep architecture recovery (27). Additionally, behavioral guidance and support provided during the intervention may have improved adherence and further contributed to sleep quality enhancement.

The lack of significant IL-6 reduction is consistent with prior studies showing that short-term dietary interventions more strongly affect CRP than IL-6, as IL-6 exhibits circadian variation and individual variability, limiting short-term decreases (28). Longer interventions (≥ 12 weeks) or greater weight loss may be required to detect significant IL-6 changes. These findings support future studies with larger sample sizes, extended intervention periods, and randomized controlled designs to rigorously validate dietary intervention effects.

Interestingly, smoking status emerged as an independent factor associated with poorer sleep outcomes, underscoring the importance of lifestyle behaviors beyond diet in shaping sleep health. This is in line with evidence that nicotine influences circadian rhythms, increases arousal, and contributes to oxidative stress, all of which may impair sleep quality. A cohort studies using objective sleep measures demonstrate that current smokers exhibit lower delta power and higher alpha power during non-REM sleep, reflecting reduced sleep depth (29). Although age and gender were not significant predictors of sleep improvement in this study, previous evidence suggests that sex hormones, body composition, and age-related metabolic changes may influence sleep regulation. The relatively homogeneous age range and predominance of middle-aged adults in our cohort may have limited the detection of such effects. Future studies with larger and more diverse populations are needed to clarify potential gender- and age-specific responses to dietary management.

### 4.1 Limitation

Despite the study provides valuable insights through its structured, individualized dietary intervention and comprehensive assessment of metabolic, inflammatory, and oxidative stress markers, contributing meaningful evidence to the field of nutritional management for metabolic syndrome. The study also has certain limitations. As a single-arm pre–post design, it cannot fully establish causal relationships between dietary intervention and observed improvements. The relatively short duration and single-center nature of the study may also limit the generalizability of the results.

## 5 Conclusion

A tailored dietary management program emphasizing energy balance, macronutrient optimization, and plant-based components significantly improved sleep quality, body weight, metabolic parameters, and inflammatory and oxidative stress markers in patients with MetS and sleep disturbances. As a safe, gentle, and effective intervention, dietary management holds promise for improving metabolic health and sleep outcomes. Future research should aim to elucidate underlying mechanisms and optimize intervention strategies.

## Data Availability

The original contributions presented in this study are included in this article/supplementary material, further inquiries can be directed to the corresponding authors.
